# Neutrophils in STAT1 Gain-Of-Function Have a Pro-inflammatory Signature Which Is Not Rescued by JAK Inhibition

**DOI:** 10.1007/s10875-023-01528-1

**Published:** 2023-06-26

**Authors:** Zuzana Parackova, Petra Vrabcova, Irena Zentsova, Anna Sediva, Marketa Bloomfield

**Affiliations:** Department of Immunology, 2nd Faculty of Medicine Charles University, University Hospital in Motol, V Uvalu 84, 515006 Prague, Czech Republic

**Keywords:** Neutrophils, STAT1 GOF, ruxolitinib, platelets, candidiasis, autoimmunity

## Abstract

STAT1 gain-of-function (GOF) mutations cause an inborn error of immunity with diverse phenotype ranging from chronic mucocutaneous candidiasis (CMC) to various non-infectious manifestations, the most precarious of which are autoimmunity and vascular complications. The pathogenesis centers around Th17 failure but is far from being understood. We hypothesized that neutrophils, whose functions have not been explored in the context of STAT1 GOF CMC yet, might be involved in the associated immunodysregulatory and vascular pathology. In a cohort of ten patients, we demonstrate that STAT1 GOF human *ex-vivo* peripheral blood neutrophils are immature and highly activated; have strong propensity for degranulation, NETosis, and platelet-neutrophil aggregation; and display marked inflammatory bias. STAT1 GOF neutrophils exhibit increased basal STAT1 phosphorylation and expression of IFN stimulated genes, but contrary to other immune cells, STAT1 GOF neutrophils do not display hyperphosphorylation of STAT1 molecule upon stimulation with IFNs. The patient treatment with JAKinib ruxolitinib does not ameliorate the observed neutrophil aberrations. To our knowledge, this is the first work describing features of peripheral neutrophils in STAT1 GOF CMC. The presented data suggest that neutrophils may contribute to the immune pathophysiology of the STAT1 GOF CMC.

**Supplementary Information:**

The online version contains supplementary material available at 10.1007/s10875-023-01528-1.

## Introduction

Chronic mucocutaneous candidiasis (CMC) refers to persistent or recurrent, non-invasive *Candida* infections of the skin, nails, and mucous membranes. STAT1 (Signal Transducer and Activator of Transcription) gain-of-function (GOF) is an inborn error of immunity, in which patients exhibit diverse phenotypes, including CMC, autoimmunity, malignancies, and vascular abnormalities, i.e., large vessel aneurysms [1–4]. While the fungal susceptibility is well attributed to the Th17 impairment, the current knowledge falls short of explaining the complex symptomatology of STAT1 GOF CMC, especially the autoimmune, vascular complications, and susceptibility to intramacrophagic pathogens.

The Janus Kinase (JAK)/STAT pathways are one of the most important cellular signaling cascades. Well over seventy different cytokines and interferons (IFN), which mediate adaptive and innate immunity, utilize this rapid membrane-to-nucleus pathway [5]. JAKs are noncovalently associated with cytokine receptors and mediate tyrosine phosphorylation of the seven STAT proteins, which dimerize and are transported into the nucleus to regulate the expression of target genes [6, 7]. Mutations in genes encoding JAK/STAT components, both loss- and gain-of function, have been found in many human diseases [6, 8]. STAT1 proteins are mainly engaged upon stimulation with type I and II IFNs and IL-27 and are involved in regulation of many processes of cellular development and functions [9–11].

In STAT1 GOF patients, the failure of antifungal immunity is attributed to Th17 impairment, resulting from dysbalanced STAT1/STAT3 signaling. The instrumentality of the augmented STAT1 phosphorylation probably includes delayed STAT1 dephosphorylation and increased amount of total STAT1 [1, 12, 13].

On the other hand, the STAT1 GOF-associated autoimmunity, affecting over one third of patients, is largely unexplained. Recently, an association with upregulated type I IFN signaling was suggested, as the autoimmune manifestations strongly resemble the clinical features of increased exposure or response to type I IFNs, characteristic for human monogenic type I interferonopathies or patients treated with recombinant IFNs [14–16]. Various STAT1 GOF cell types were demonstrated to exhibit increased IFN-induced STAT1 phosphorylation and/or increased type I IFN signature [1, 13].

Surprisingly little is known about the consequences of hyperactivating *STAT1* mutation in non-lymphoid cells, particularly those involved in innate immune functions. Recently, we demonstrated that STAT1 GOF monocytes and dendritic cells display proinflammatory features and impaired regulatory functions [17, 18]. No reports have yet addressed the role of neutrophils in STAT1 GOF, perhaps because isolated neutropenia only rarely causes CMC [19]. However, neutrophils and their products have been implicated in various human autoimmune diseases, such as systemic lupus erythematodes (SLE), psoriasis, or type I diabetes mellitus (T1D) [20]; therefore, an investigation of their role in STAT1 GOF is warranted.

Here, we performed phenotypical, functional, and transcriptomic studies of *ex vivo* peripheral neutrophils from STAT1 GOF patients and from STAT1 GOF patients treated with Janus kinase inhibitor (JAKinib) ruxolitinib (RUXO) to elucidate their role in the disease-associated immunodysregulatory phenomena.

## Patients and Methods

### The Patient Cohort Characteristics

**Fig. 1:**
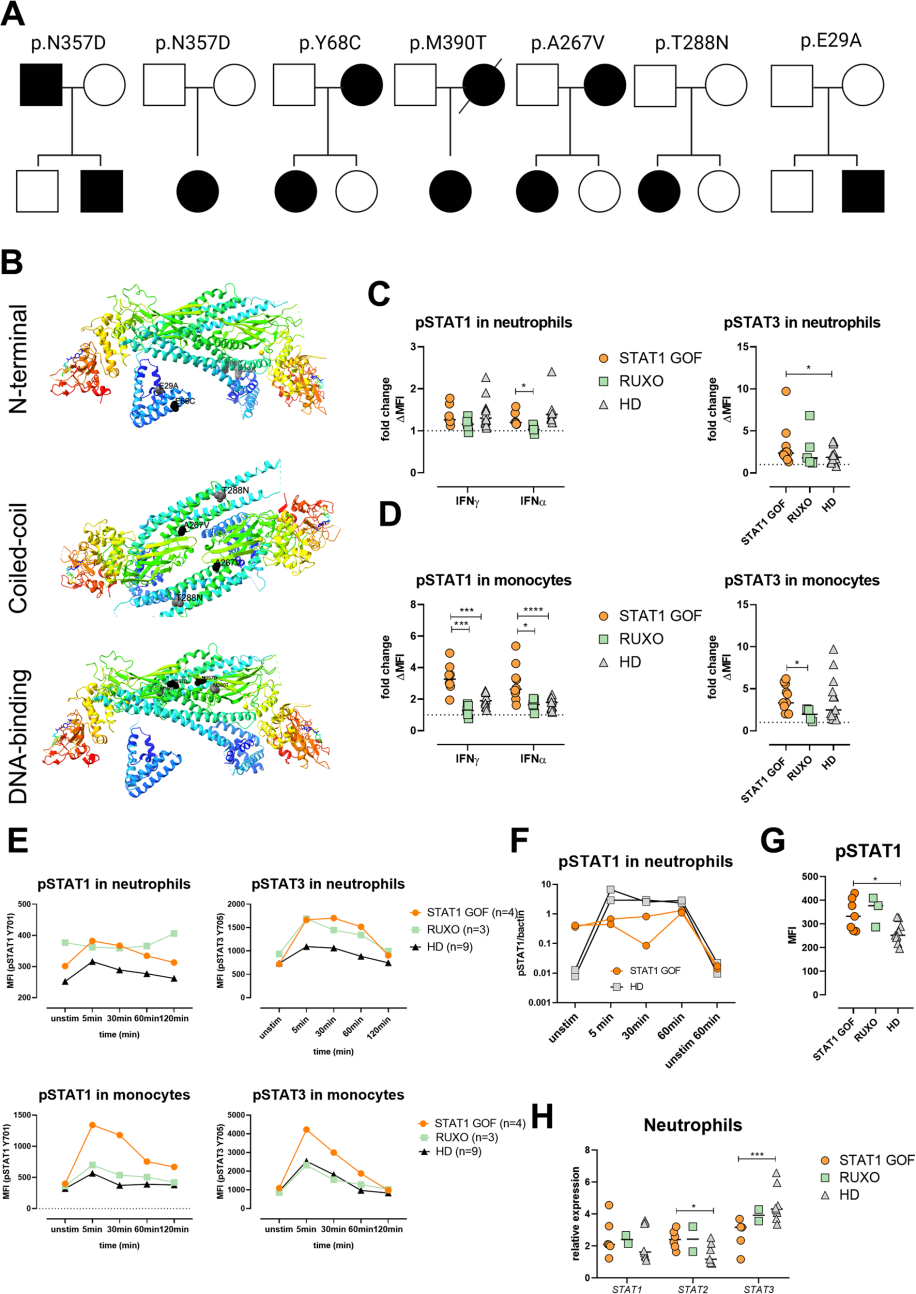
Patients and STAT1 mutations. **A** Pedigrees of STAT1 GOF mutations in individual kindreds. Mutation carriers are displayed in black. **B** STAT1 protein 3D structure with highlighted mutation positions. **C** Phosphorylation of pSTAT1 (Tyr701) and pSTAT3 (Tyr705) in neutrophils upon IFNα and IFNγ stimulation for 5 minutes in STAT1 GOF patients (*n*=8), RUXO patients (*n*=5), and HD (*n*=16). Data are expressed as fold change of stimulated/unstimulated samples. **D** Phosphorylation of pSTAT1 (Tyr701) and pSTAT3 (Tyr705) in monocytes upon IFNα and IFNγ stimulation for 5 minutes in STAT1 GOF patients (*n*=8), RUXO patients (*n*=5), and HD (*n*=16). Data are expressed as fold change of stimulated/unstimulated samples. **E** Kinetics of STAT1 and STAT3 phosphorylation upon IFNα stimulation in neutrophils and monocytes detected in STAT1 GOF patients (*n*=4), RUXO patients (*n*=3), and HD (*n*=10) by flow cytometry **F.** Semiquantitative analysis of pSTAT1/βactin using ImageJ software. **G** pSTAT1 MFI quantification in unstimulated STAT1 GOF patients (*n*=7), RUXO patients (*n*=3), and HD (*n*=9) detected by flow cytometry. **H** *STAT1*, *STAT2*, and *STAT3* gene relative expression detected in STAT1 GOF patients (*n*=6), RUXO patients (*n*=2), and HD (*n*=8) by real-time PCR. RUXO, ruxolitinib-treated patients; HD, healthy donors; Tyr, tyrosine; ND, N-terminal domain; CCD, coiled-coil domain; DBD, DNA-binding domain; LD, linker domain; SH2, Src homology 2 domains; TAD, tyrosine activation domain and a transcriptional activation domain; MFI, mean intensity fluorescence. Values are standardized and expressed as median values. Statistical analyses were performed using paired *t*-tests. Values of *p*<0.05 (*), *p*<0.01 (**), *p*<0.001 (***), and *p*<0.0001 (****) were considered statistically significant

Ten patients, three male and seven female, from seven non-consanguineous Czech families of Caucasian ethnicity were included in this study. Eight of the patients were reported by us previously [17, 18]. All patients had genetically confirmed *STAT1* GOF mutation, detected either by Sanger sequencing or by next generation sequencing of in-house panel of selected inborn error of immunity-associated genes or whole exome sequencing (Fig. 1A). The patients harbored known heterozygous mutations affecting the N-terminal (p.E29A, p.Y68C), coiled-coil (p.A267V, p.T288N), and DNA-binding (p.N357D, p.M390T) protein domains (Fig. 1B), and all had detectable hypersignaling downstream from IFNα- and/or IFNγ-recruited STAT1 pathway in T lymphocytes/monocytes. All patients suffered from CMC of various severity, and all had increased infectious susceptibility to bacterial or viral pathogens. Four patients had clinically manifest autoimmunity, and seven patients had detectable autoantibodies against various, predominantly organ-nonspecific antigens. One patient had aortic aneurysm (P6); no malignancy was diagnosed in the cohort. All patients received antifungal prophylaxis, three patients were treated with selective JAK 1/2 inhibitor ruxolitinib—P1 for CMC and multiple autoimmune features, P2 for refractory CMC and severe keratitis and P10 for refractory CMC and severe lung disease (samples from P2 and P10 were obtained prior and after ruxolitinib initiation, samples from P1 were obtained on ruxolitinib only).

All but two patients had decreased peripheral CD4+ Th17 lymphocyte count, while the rest of the T cell pool was unaffected in the majority of the cohort. Three patients had low circulating numbers of mature B cells, and all but one patient displayed some degree of dysgammaglobulinemia. No patient had neutropenia at the time of sample acquisition. The summary of genotypes, clinical phenotypes, and an overview of routine immunological investigations is available in Tables 1 and 2.

**Table 1:** Genetic and clinical characteristics of STAT1 GOF patients. Expanded from Parackova et al. Clinical Immunology, 2022 [18]

	cDNA position	Protein domain	M/F (gender identity)	Age (years)	CMC	Other infections	Autoimmunity	Autoantibodies	Pneumopathy	Aneurysms/malignancy	Others	Treatment
P1^E29A^	c.86A>C	N-terminal	M (M)	47	**+**	SPI	AIHA, TH, AIH, CD	Coombs, a-TTG, a-EM, a-TG	BE	−/−	AS, KT, ↑LFT	AZ, CS, RTX, RX,
P2^Y68C^	c.203A>G	N-terminal	F (F)	24	**+**	SPI	−	ASCA	−	−/−	AS, KT, ↑LFT, VC	AZ, RX, IgRT
P3^Y68C^	c.203A>G	N-terminal	F (F)	47	**+**	SPI	TH	ASCA, ANA, a-GLD	BA	−/−	AS, KT, ↑LFT, VC	AZ
P4^A267V^	c.800C>T	Coiled-coil	F (F)	48	**+**	SPI HSV	TH	ASCA	−	−/−	KT, CG, OeS, VC	AZ, TMP
P5^A267V^	c.800C>T	Coiled-coil	F (F)	22	**+**	OMA	TH	ASMA	−	−/−	CDu, CD	AZ, TMP
P6^T288N^	c.863C>A	Coiled-coil	F (F)	52	+	SPI HSV	−	ASCA, CCP, AECA	MVI	Aortal aneurysm−	AS, KT, ↑LFT, VC	AZ
P7^N357D^	c.1069A>G	DNA-binding	M (M)	8	**+**	OMA	−	−	−	−/−	−	AZ
P8^N357D^	c.1069A>G	DNA-binding	M (M)	45	**+**	HSV	−	ASCA, RF	−	−/−	−	AZ
P9 ^N357D^	c.1069A>G	DNA-binding	F (F)	11	**+**	OMA, VZV,	−	−	−	−/−	VC	AZ
P10^M390T^	c.1169T>C	DNA-binding	F (F)	20	**+**	SPI VZV	−	−	BE, AT, MVI	−/−	AS, KT	AMP, AZ, RX, IgRT

*a-AECA* anti-endothelial cell antibodies, *a-CCP* anti-cyclic citrulinated peptide, *a-EM* anti-endomysium, *a-GLD* anti-gliadin antibodies, *a-TG* anti-thyreoglobulin, *a-TTG* anti-thyreoglobulin, *AIH* autoimmune hepatitis, *AIHA* autoimmune haemolytic anemia, *ANA* anti-nuclear antibodies, *AS* aphthous stomatitis, *ASCA* anti-saccharomyces cerevisiae, *AT* atelectases, *BA* bronchial asthma, *BE* bronchiectasis, *CD* celiac/celiac like disease, *CDu* chronic duodenitis, *CG* chronic gastritis, *CMC* chronic mucocutaneous candidiasis, *HSV* herpes simplex virus, *KT* keratitis, LFT liver function tests, *M/F* male/female, *MVI* mixed ventilatory impairment, *OeS* esophageal strictures, *OMA* otitis media, *RF* rheumathoid factor, *SPI* sinopulmonary infections, *TH* thyroiditis, *VC* vaginal candidiasis, *VZV* varicella zoster virus

Treatment: *AMP* amphotericin B, *AZ* azoles, *CS* corticosteroids, *IgRT* immunoglubulin replacement therapy, *RTX* rituximab, *RX* ruxolitinib, *TMP* trimethoprim

**Table 2:** Immunologic characteristics of STAT1 GOF patients. Expanded from Parackova et al. Clinical Immunology, 2022 [18]

	IgG* (g/L)	IgG1* (g/L)	IgG2* (g/L)	IgG3* (g/L)	IgG4* (g/L)	IgA* (g/L)	IgM* (g/L)	Specific antibodies*	CD4/CD8 T cells° (10^9^/L)	Th17° (%IL17+ of CD4+)	B cells° (10^9^/L)	Neutrophil count° (10^9^/L)	Trombocyte count° (10^9^/L)	CRP° (mg/L)	pSTAT1° (Tyr701)
P1^E29A^	9.41 (7.65–13.60)	7.83 g/L (4.90–11.40)	↓ 0.84 (1.50–6.40)	0.72 (0.20–1.10)	↓ <0.07 (0.08–1.40)	2.43 (0.91–2.90)	1.43 (0.47–1.95)	•↓ Tetanus 0.04 IU/mL (>0.10) •↓ COVID-19 mRNA vaccine IgG 5.6 U/mL (>18.0)	Normal	↓ 0.16 (0.40–1.80)	Not detected–post rituximab	4.66 (2.00–7.00)	210 (150–450)	↑ 15.3 (0.0–8.0)	↑
P2^Y68C^	12.30 (7.65–13.60)	8.44 (4.90–11.40)	4.33 (1.50–6.40)	↑ 1.19 0.20–1.10	0.17 (0.08–1.40)	↑ 4.27 (0.91–2.90)	↓ 0.42 (0.47–1.95)	•Tetanus 0.2 IU/mL (>0.10) •Measles 214 mIU/mL (>200) •COVID-19 mRNA vaccine IgG >1000 U/mL (>18)	Normal	1.4 (0.40–1.80)	Normal	2.56 (2.00–7.00)	315 (150–450)	↑ 10.9 (0.0–8.0)	↑
P3^Y68C^	↑ 14.80 (7.65–13.60)	↑ 12.40 (4.90–11.40)	4.25 (1.50–6.40)	0.81 (0.20–1.10)	↑ 2.44 (0.08–1.40)	↑ 4.34 (0.91–2.90)	0.52l (0.47–1.95)	•Tetanus 1.2 IU/mL (>0.10) •Measles 693 mIU/mL (>200) •COVID-19 mRNA vaccine IgG >1000 U/mL (>18.0)	Normal	0.88 (0.40–1.80)	↓ CD27+ memory	3.05 (2.00–7.00)	263 (150–450)	↑ 10.6 (0.0–8.0)	↑
P4^A267V^	↑ 18.80 (7.65–13.60)	↑ 12.60 (4.90–11.40)	3.39 (1.50–6.40)	↑ 2.43 (0.20–1.10)	↓ <0.07 (0.08–1.40)	↓ <0.07 (0.91–2.90)	0.58 (0.47–1.95)	•Tetanus 1.91 IU/mL (>0.10) •COVID-19 mRNA vaccine IgG 855.7 U/mL (>18.0)	Normal	↓ 0.35 (0.40–1.80)	Normal	3.29 (2.00–7.00)	295 (150–450)	1.9 (0.0–8.0)	↑
P5^A267V^	11.60 (7.65–13.60)	8.99 (4.90–11.40)	2.23 (1.50–6.40)	0.58 (0.20–1.10)	↓ <0.07 (0.08–1.40)	↑ 3.13 (0.91–2.90)	0.75 (0.47–1.95)	•Tetanus 0.14 IU/mL (>0.10) •COVID-19 mRNA vaccine IgG >1000.0 U/mL (>18.0)	Normal	↓ 0.24 (0.40–1.80)	Normal	2.41 (2.00–7.00)	263 (150–450)	5.4 (0.0–8.0)	↑
P6^T288N^	9.08 (7.65–13.60)	6.22 (4.90–11.40)	1.62 (1.50–6.40)	↑ 1.51 (0.20–1.10)	↓ <0.08 (0.08–1.40)	2.48 (0.91–2.90)	0.50 (0.47–1.95)	•Tetanus 0.38 IU/mL (>0.10) •↓ Measles <50 mIU/mL (>200) •COVID-19 mRNA vaccine IgG >1000 U/mL (>18)	↓ CD4 0.26 (0.30-2.80) Otherwise normal	↓ 0.39 (0.40–1.80)	↓ CD27+ memory ↓ Class switched	3.49 (2.00–7.00)	↓ 97 (150–450)	↑ 9.0 (0.0–8.0)	↑
P7^N357D^	7.31 (6.37–11.05)	5.24 (3.70–10.00)	1.79 (0.72–3.40)	0.40 (0.13–1.33)	<0.07 (0.01–1.58)	0.66 (0.58–1.16)	0.49 (0.47–1.67)	•Tetanus 1.44 IU/mL (>0.10) •HiB > 9 IU/mL (>0.10)	Normal	↓ 0.33 (0.40–1.80)	↑ IgD− CD27− naive	2.87 (1.90–9.70)	333 (150–450)	3.0 (0.0–8.0)	↑
P8^N357D^	10.70 (7.65–13.60)	7.16 (4.90–11.40)	4.40 (1.50–6.40)	0.61 (0.20–1.10)	0.43 (0.08–1.40)	↑ 3.06 (0.91–2.90)	1.23 (0.47–1.95)	•↓ Tetanus 0.08 IU/mL (>0.10) •↓ Measles <50 mIU/mL (>200)	Normal	↓ 0.34 (0.40–1.80)	Normal	4.99 (2.00–7.00)	222 (150–450)	1.2 (0.0–8.0)	↑
P9^N357D^	↑ 16.50 (6.37–11.05)	11.37 (4.90–11.40)	2.71 (0.72–3.40)	0.54 (0.13–1.33)	<0.07 (0.01–1.58)	1.28 (0.79–1.37)	1.05 (0.47–1.73)	•↓ Tetanus 0.03 IU/mL (>0.10)	Normal	↓ 0.20 (0.40–1.80)	↓ CD27+ memory ↓ Class switched	2.93 (1.90–9.10)	273 (150–450)	2.7 (0.0–8.0)	↑
P10^M390T^	↑ 16.50 (7.31–12.75)	↑ 13.08 (4.00–11.50)	1.49 (0.98–4.80)	↑ 1.55 (0.15–1.49)	<0.07 (0.03–2.10)	0.94 (0.91–2.90)	0.61 (0.47–1.95)	•Tetanus 0.35 IU/mL (>0.10) •↓ Measles 135 mIU/mL (>275) •COVID-19 mRNA vaccine IgG 329.9 U/mL (>18)	↓ CD4 0.54 (0.56-2.70) Otherwise normal	↓ 0.07 (0.40–1.80)	Normal	4.47 (2.00–7.00)	331 (150–450)	↑ 11.3 (0.0–8.0)	↑

* prior to immunoglobulin replacement therapy, where applicable; *°* prior to ruxolitinib, where applicable; *pSTAT1* IFNγ/IFNα-induced phosphorylation of p-STAT1 (Tyr701) in CD3^+^ T lymphocytes, *CRP* C-reactive protein, *HiB* Heamophilus Influenzae type B, *↑* increased, *↓* decreased

The patients are followed at the Department of Immunology in University Hospital in Motol, 2nd Faculty of Medicine, Charles University in Prague. The study was carried out in accordance with the recommendations of the institutional Ethical Committee; the study protocol was approved by the Ethical Committee. All subjects or their legal guardians gave written informed consent in accordance with the Declaration of Helsinki.

### Phosphoflow

Whole blood was stimulated with 1μg/ml IFNγ or IFNα (Abcam, Cambridge, UK) for 5, 30, 60, and 120 minutes or left untreated at 37°C. Intracellular signaling was prevented by using 4% paraformaldehyde without methanol for 10 minutes at room temperature. Erythrocytes were lysed using 0.1% Triton-X for 20 minutes (Sigma Aldrich, St. Luis, USA) at 37°C; leukocytes were permeabilized with ice-cold 80% methanol for 30 minutes and stained with anti-phosphoSTAT1-BV421 (Tyr701) (clone 4a) and anti-phosphoSTAT3-PE (Tyr705) (clone 4/5-STAT3) (both from BD Bioscience, San Jose, USA), anti CD14-APC (63D3), CD66b-PC7 (clone G10F5) (BioLegend, San Diego, USA), and anti CD3-A700 (Exbio, Vestec, Czech Republic). The samples were acquired on BD Fortessa (BD Biosciences), and data analysis was performed using FlowJo (TreeStar).

### STAT1 Graphics

Molecular graphics performed with UCSF ChimeraX, developed by the Resource for Biocomputing, Visualization, and Informatics at the University of California, San Francisco, with support from National Institutes of Health R01-GM129325 and the Office of Cyber Infrastructure and Computational Biology, National Institute of Allergy and Infectious Diseases [21].

### Western Blot

Neutrophils were stimulated for 5, 30, and 60 minutes with IFNα or left untreated, washed, and lysed in RIPA lysis buffer and PMSF (CellSignaling, Danvers, USA), placed on ice, sonicated, and then centrifuged at 14000 g to remove cell debris. For western blot analysis, samples were resuspended in Laemmli buffer (Sigma Aldrich) at 1:1 ratio and boiled for 5 min. Proteins were separated by SDS-PAGE, transferred to the PVDF membrane. After blocking with 5% BSA for 2 hours in TBST (TBS and 0,1%Tween, both from Bio-Rad, Hercules, USA), membranes were incubated with the primary antibodies anti pSTAT1 (M135), anti STAT1 (9H2), and βactin (D6A8) (CellSignalling) overnight, followed by incubation with peroxidase-conjugated anti rabbit or anti mouse secondary antibodies for 2 hours. Membranes were developed using SuperSignal West Femto (Thermo Fisher Scientific). Densitometry was performed with ImageJ software (National Institutes of Health, USA). Band area values were used for semi-quantification. Graphs are expressed as ratio of stimulated/unstimulated cells of band area value calculated from band area of phosphorylated forms/band area of βactin.

### RNA Isolation and Gene Expression

Total RNA was isolated using the RNeasy Mini kit following the manufacturer’s instructions (Qiagen, Hilden, Germany). RNA quality and quantification were determined by TapeStation 4200 (Agilent, St. Clara, USA) following the manufacturer’s instructions. Neutrophil total RNA was isolated using RNeasy Mini Kit following the manufacturer’s instructions (Qiagen), and complementary DNA (cDNA) was synthesized using M-MLV Reverse Transcriptase (Thermo Fisher Scientific). RT-PCR was performed in duplicates using the cDNA and Platinum Taq polymerase (Thermo Fisher Scientific), 200 nM dNTP (Promega, Southampton, UK), 50mM MgCl_2_ (Thermo Fisher Scientific), and TaqMan primer/probe sets (Thermo Fisher Scientific). Samples were matched to a standard curve generated by amplifying serially diluted products using the same PCR reaction and normalized to *GAPDH* (Hs00266705_g1) to obtain the relative expression value. Real-time assays were run on FX96 Cycler (Bio-Rad). The following are the primes used: *STAT1* (Hs01013996_m1), *STAT2* (Hs01013115_g1), *STAT3* (Hs00374280_m1), *IFIH1* (Hs00223420_m1), *IFIT* (Hs00356631_g1), *ISG15* (Hs00192713_m1), *MX1* (Hs00895608_m1), *IRF1* (Hs00971965_m1), *SOCS1* (Hs00705164_s1), *USP18* (Hs00276441_m1), *IFI44* (Hs00197427_m1), *IFI16* (Hs00986757_m1), and *OAS* (Hs00242943_m1) (all from Thermo Fisher Scientific).

### NanoString Analysis

Approximately 50 ng of total RNA from neutrophils was used to measure the expression of 730 myeloid immunity-related genes and 40 housekeeping genes using the nCounter platform (NanoString Technologies) and the Myeloid Innate immunity panel. Data were log base 2-transformed and normalized using housekeeping genes in nSolver. For analysis Rosalind and Enrichr analysis platform and BioPlanet 2019, a tool that integrates pathway annotations from publicly available curated sources was used.

### Cell Isolation and Culture

Peripheral blood was collected from patients and healthy volunteers into EDTA-coated tubes. First, peripheral blood mononuclear cells (PBMCs) were isolated using Ficoll-Paque (GE Healthcare Biosciences, Uppsala, Sweden). Neutrophils (polymorphonuclear leukocytes, PMNs) were further isolated using the Dextran sedimentation method. The obtained neutrophils were resuspended in RPMI 1640 medium supplemented with 10% fetal bovine serum, 1% penicillin, and streptomycin and 1% Glutamax (Thermo Fisher Scientific, Waltham, USA), seeded in 96-well plates (Thermo Fisher Scientific) at 1×10^6^/ml concentration and stimulated with 100ng/ml LPS, 100ng/ml zymosan, or *C. albicans* for 24 hours, and then cytokine levels in cell-free supernatants were determined by multiplex Luminex cytokine bead-based immunoassay (Merck Millipore, Bedford, USA).

### Neutrophil Phenotype and Subsets

Peripheral blood was stained with a mixture of antibodies containing anti-lineage specific markers (CD3 clone MEM-57, CD19 clone LT19, CD20 clone LT20, CD56 clone MEM-188, CCR3 clone 5E8)-FITC, CD10-PEDY594 (clone MEM-78) (Exbio, Prague, Czech Republic), CD14-APC (clone HDC14), CD66b-PC7 (clone G10F5), CD62L-BV650 (clone DREG-56), CD11b-BV510 (clone ICRF44), CD16-A700 (clone 3G8), CD33-BV421 (clone P67.6), PDL1-PE (clone 29E.2A3) (Biolegend, San Diego, CA, USA), and HLA-DR-PerCP (clone 243) (BD Biosciences, San Jose, CA, USA) for 20 minutes and then hypotonically lysed. Suppressive neutrophils were defined as CD66b+ CD62L^lo^CD16+ and immature neutrophils as CD10-CD66b+CD16+. G-MDSC subset was defined as CD33^hi^CD11b+CD16+CD66b+ neutrophils. Aged neutrophils were determined as CXCR4+ CD62L− subpopulation after staining peripheral blood with a mixture of antibodies containing CXCR4-PE (clone 12G5), CXCR2-FITC (clone 5E8), CD66b-PC7 (clone G10F5), CD62L-BV650 (clone DREG-56) (Biolegend), CD10-PEDY594 (clone MEM-78), and CD16-A700 (3G8). The gating strategies are shown in Supplementary Figure 1A. Samples were acquired on BD Fortessa and analyzed using FlowJo software.

For tSNE analysis, expression of CD66b, CD11b, CD16, HLA-DR, PD-L1, CD62L, and CD10 was used. Itineration was set to 1000, perplexity to 30, and learning rate to 4380.

### Serum Product Determination

Various ELISAs were used for detection of serum levels of S100A8/A9, myeloperoxidase (MPO), neutrophil elastase (NE), CXCL8, VCAM-1, ICAM-1, CD62L (Abcam), and DNA-histone complexes (Merck Millipore). For CXCL4, lipocalin, lactoferrin, MMP8, proteinase 3, sCD62P, and sCD40L were determined by LUMINEX xMAP Technology.

### Phagocytosis

The capacity to phagocyte zymosan (Green Zymosan) and *E. coli* (Red *E. coli*) was determined using commercially available kits from Abcam. In brief, cells were incubated for 2 hours with fluorescently labeled zymosan or 30 minutes with fluorescently labeled *E. coli*, respectively, stained with CD66b-FITC or PE-Cy7 respectively for 30 minutes, hypotonically lysed, washed, and analyzed by flow cytometry.

### ROS Production

The release of reactive oxygen species (ROS) was determined using commercially available kit from Exbio upon *E. coli* and PMA stimulation by flow cytometry. The heparinized blood was stimulated for 30 minutes in presence of dihydrorhodamine 123 (DHR123). The kit detects fluorescent rhodamine 123 as a result of NADPH oxidase activation.

### NETosis Assay

The neutrophils were seeded in black flat 96-well plates at 0.5×10^6^/ml concentration and stimulated with 50ng/ml PMA 30 minutes. A cell impermeable DNA binding Sytox Green dye (Thermo Fisher Scientific) was used for measuring the real-time kinetics of NET release. Neutrophils were mixed with 1μM Sytox Green dye, and the changes in green fluorescence signal were measured every 30 minutes using a fluorescence plate reader Synergy H1 (Agilent). Each condition was tested with a technical duplicate.

### Platelet-Neutrophil Aggregates

Peripheral blood was stained with a mixture of antibodies containing anti-lineage specific markers (CD3 clone MEM-57, CD19 clone LT19, CD20 clone LT20, CD56 clone MEM-188, CCR3 clone 5E8)-FITC, CD14-APC (clone HDC14), CD66b-PC7 (clone G10F5), CD15-A700 (clone W6D3), CD41-PE (clone MEM-06), and CD62P-BV650 (clone AK4) (Biolegend) for 20 minutes and then hypotonically lysed. Samples were acquired on FACSFortessa and analyzed using FlowJo software.

### Quantification and Statistical Analysis

The results obtained from at least three independent experiments are given as the medians±SDs. Not all patients were involved in all experiments due to the limited amount of blood available per sample. Statistical analysis was performed using non-parametric one-way analysis of variance (ANOVA) with multiple comparisons Dunn’s post-test where applicable. A two-tailed paired Wilcoxon or unpaired Mann-Whitney *t*-test was also applied for data analysis using GraphPad Prism 8. Values of *p*<0.05 (*), *p*<0.01 (**) *p*<0.001 (***), and *p*<0.0001 (****) were considered statistically significant.

## Results

### STAT1 Hyperphosphorylation Does Not Manifest in STAT1 GOF Neutrophils

The characteristic feature of STAT1 GOF CMC is the increased STAT1 phosphorylation upon IFNγ, IFNα, or IL-27 stimulation in T and B lymphocytes, monocytes, or dendritic cells [17, 18, 22]. Accordingly, we stimulated peripheral blood with IFNα or IFNγ for 5 minutes and analyzed STAT1 and STAT3 phosphorylation in healthy donor (HD) and patient neutrophils. Interestingly, no differences in phosphorylated STAT1 (pSTAT1) upon stimulation were noted between the cohorts (Fig. 1C and Supplementary Figure 1A). However, a higher IFNα-induced STAT3 phosphorylation (pSTAT3) was detected in patient neutrophils (Fig. 1C). Moreover, neutrophils from patients treated with RUXO demonstrated only minor changes in pSTAT1 and pSTAT3 compared to untreated patients. To exclude a technical error, we assessed IFN-induced pSTAT1 and pSTAT3 levels in monocytes from the same samples (Fig. 1D) and detected markedly increased pSTAT1, but not pSTAT3 in the patients’ samples, correlating well with the previously published observations.

Next, we stimulated whole blood with IFNα for 5, 30, 60, and 120 minutes and analyzed pSTAT1 and pSTAT3 kinetics in neutrophils, along with monocytes as a control cell population. In patient neutrophils, pSTAT1 mirrored the kinetics of HD cells (Fig. 1E), while in patient monocytes the dephosphorylation was delayed compared to HD. In RUXO-treated patient neutrophils, higher unstimulated pSTAT1 was detected, and IFNα-stimulated pSTAT1 kinetics was comparable to patients without RUXO treatment. A similar level of pSTAT3 was found in all patient neutrophils, regardless of the treatment, and it was increased compared to HD (Fig. 1E). Levels of pSTAT1 and pSTAT3 in RUXO treated patients’ monocytes were comparable to HD (Fig. 1E).

These unexpected findings were affirmed with western blot. The quantitative analysis of pSTAT1 to β actin expression ratio in samples confirmed similar level of phosphorylation in HD and STAT1 GOF neutrophils (Fig. 1F and Supplementary Figure 1B-D) upon IFNα stimulation. Intriguingly, the western blot demonstrated increased STAT1 phosphorylation also in unstimulated patient neutrophils (Fig. 1F). This observation was confirmed by flow cytometry (Fig. 1G).

To assess whether the comparable pSTAT1 between patients and HD might be due to altered STAT1 expression in neutrophils, we performed real-time PCR in isolated neutrophils, detecting RNA levels of *STAT1*, *STAT2*, and *STAT3* molecules. No differences in *STAT1* expression were noted; however, patient neutrophils expressed increased levels of *STAT2* and decreased levels of *STAT3* (Fig. 1H).

### Cytokine Signaling Is the Main Distinction Between Healthy and STAT1 GOF Neutrophils

**Fig. 2:**
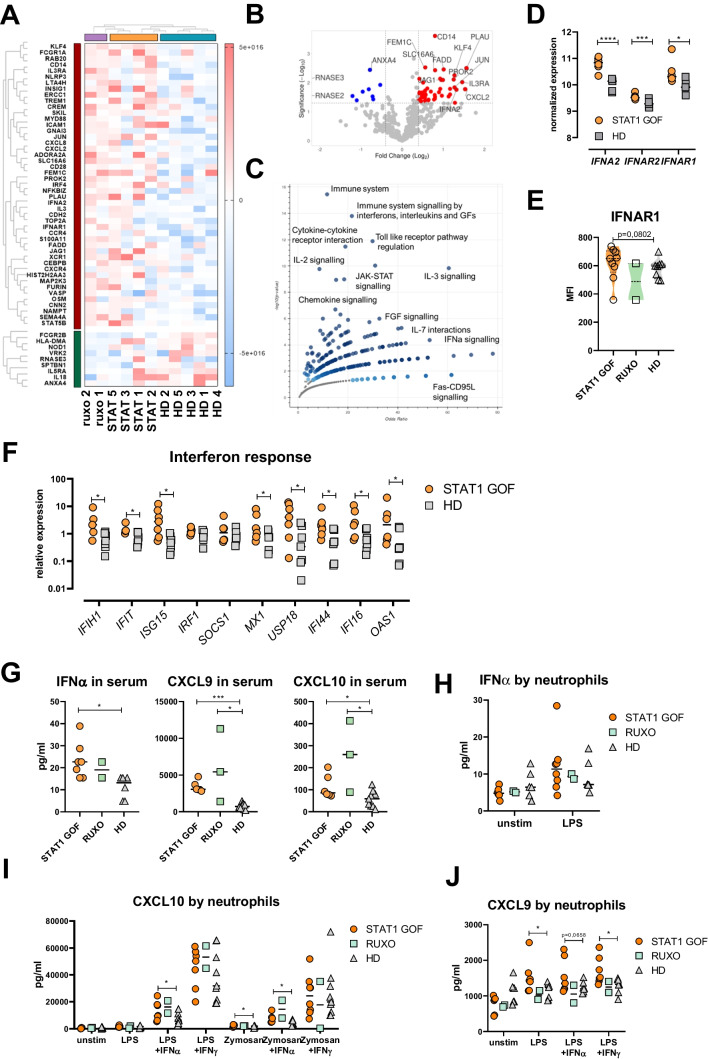
STAT1 GOF neutrophil transcriptomic analysis and interferon response. **A** Heatmap of differentially expressed genes (DEGs) in STAT1 GOF (*n*=4), RUXO (*n*=2), and HD (*n*=5) neutrophils. **B** Volcano plot of up- and downregulated DEGs. **C** Volcano plot of terms in the Bioplanet_2019 gene set library. Each point represents a single term in the library, plotted by the corresponding odds ratio (x-position) and −log10 (p-value) (y-position) from the enrichment DEG results. **D** *IFNA*, *IFNAR2*, and *IFNAR1* normalized expression in neutrophils of STAT1 GOF (*n*=6) and HD (*n*=5) detected by nanoString platform. **E** IFNAR1 expression on neutrophil surface in STAT1 GOF patients (*n*=9), RUXO patients (*n*=2), and HD (*n*=9) detected by flow cytometry. **F** Relative expression of interferon-induced genes in STAT1 GOF patients (*n*=8) and HD (*n*=8) neutrophils detected by real-time PCR. **G** Serum levels of IFNα, CXCL9, and CXCL10 in STAT1 GOF patients (*n*=6), RUXO patients (*n*=2), and HD (*n*=10). **H** IFNα production by neutrophils in STAT1 GOF patients (*n*=8), RUXO patients (*n*=2), and HD (*n*=6) upon LPS stimulation. **I** CXCL10 production by neutrophils in STAT1 GOF patients (*n*=7), RUXO patients (*n*=2), and HD (*n*=9) upon LPS, zymosan stimulation, and their combination with IFNα or IFNγ. **J** CXCL9 production by neutrophils in STAT1 GOF patients (*n*=7), RUXO patients (*n*=2), and HD (*n*=9) upon LPS stimulation and its combination with IFNα or IFNγ overnight. DEG, differentially expressed gene; HD, healthy donors; IFN, interferon; LPS, lipopolysaccharide; RUXO, ruxolitinib-treated patients. Values are standardized and expressed as median values. Statistical analyses were performed using paired *t*-tests. Values of *p*<0.05 (*), *p*<0.01 (**), *p*<0.001 (***), and *p*<0.0001 (****) were considered statistically significant

Despite the normal STAT1 phosphorylation, we hypothesized that neutrophil features would be affected by STAT1 mutation. Therefore, using nCounter Myeloid Innate Immunity panel, containing 770 genes, we performed transcriptomic analysis of patient neutrophils. Out of 770 analyzed genes, 57 genes distinguished STAT1 GOF from HD neutrophils (Fig. 2A, B). These differentially expressed genes (DEGs) are involved mostly in signaling cascades of Toll-like receptors (TLRs) and various cytokines, such as IL-2 IL-3, IL-7, and IFNs (*IFNΑ2*, *IFNAR1*, and *IFNAR2*) and, as expected, in JAK-STAT pathway (Fig. 2C). For transcriptomic analysis on RUXO-treated patients (Supplementary Figure 3), see below.

### Interferon Response Is Enhanced in STAT1 GOF Neutrophils

The increased expression of *IFNΑ2*, *IFNAR1*, and *IFNAR2* in STAT1 GOF neutrophils was interesting (Fig. 2D) as neutrophils are not typically associated with robust IFN response. Therefore, we first investigated IFNAR1 expression on neutrophil surface by flow cytometry and found it only slightly increased compared to HD (statistically not significant; Fig. 2E). Then, using real-time PCR, we detected expression of genes related to IFN response and found 8 out of 10 detected genes to be significantly increased in the patient neutrophils (Fig. 2F).

Next, we measured serum level of IFNα in the patient samples and found it increased (Fig. 2G). Similarly, increased levels of other IFN response-related chemokines, CXCL10 and CXCL9, were found (Fig. 2G and Supplementary Figure 2A). Conversely, the production of IFNα by patient neutrophils compared to HD was unincreased (Fig. 2H). Likewise, the spontaneous release of CXCL9 and CXCL10 by neutrophils was unincreased, although their production was increased upon co-stimulation with lipopolysaccharide (LPS) or zymosan with IFNs (Fig. 2I, J).

### Ruxolitinib Does Not Ameliorate the Transcriptomic Differences Between STAT1 GOF and Healthy Neutrophils

82 DEGs that differed between RUXO and HD neutrophils were detected (Supplementary Figure 3), which even exceeded the number when untreated patient neutrophils were compared to HD. These genes are involved in basic neutrophil biology, i.e., degranulation, activation, and neutrophil-mediated immune response (Supplementary Figure 3C). Similar to STAT1 GOF neutrophils, other affected biological processes were cytokine signaling, namely, IL-2, IL-8, TLR pathways, LPS-induce responses, and regulation of T helper cells (Supplementary Figure 3D).

### Neutrophil Degranulation Products Are Increased in STAT1 GOF Sera and Supernatants

**Fig. 3:**
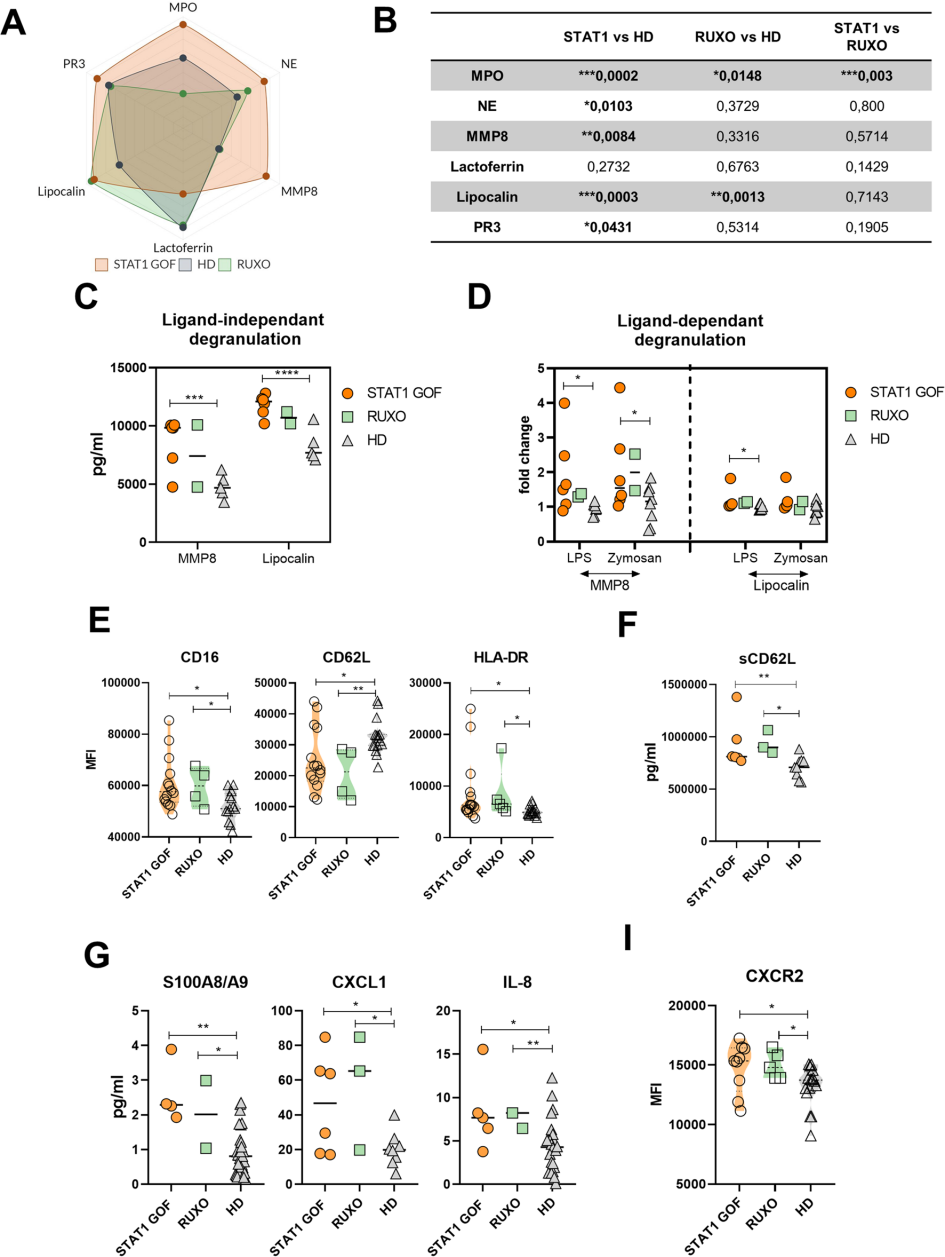
STAT1 GOF neutrophil degranulation and activation. **A** Radar plot of serum degranulation products in STAT1 GOF (*n*=6), RUXO (*n*=3), and HD (*n*=10). **B** Table with *p*-values. **C** Ligand-independent release of MMP8 and lipocalin of neutrophils in STAT1 GOF (*n*=6), RUXO (*n*=2), and HD (*n*=5) detected by Luminex. **D** Ligand-dependent release of MMP8 and lipocalin by neutrophils upon LPS and zymosan stimulation in STAT1 GOF (*n*=6), RUXO (*n*=2), and HD (*n*=5) detected by Luminex. **E** Expression of CD16, CD62L, and HLA-DR on neutrophil surface in STAT1 GOF (*n*=15), RUXO (*n*=5), and HD (*n*=17) detected by flow cytometry. **F** Serum level of CD62L in STAT1 GOF (*n*=6), RUXO (*n*=3), and HD (*n*=10) detected by Luminex. **G** Serum level of S100A8/A9, CXCL1, and IL-8 in STAT1 GOF (*n*=6), RUXO (*n*=3), and HD (*n*=25) detected by Luminex. **I** Expression of CXCR2 on neutrophil surface in STAT1 GOF (*n*=9), RUXO (*n*=5), and HD (*n*=16) detected by flow cytometry. LPS, lipopolysaccharide; HD, healthy donors; RUXO, ruxolitinib-treated patients. Values are standardized and expressed as median values. Statistical analyses were performed using paired *t*-tests. Values of *p*<0.05 (*), *p*<0.01 (**), *p*<0.001 (***), and *p*<0.0001 (****) were considered statistically significant

To follow up on transcriptomic data, we examined neutrophil degranulation markers in patient sera, namely, myeloperoxidase (MPO), neutrophil elastase (NE), matrix metalloproteinase 8 (MMP8), lactoferrin, lipocalin, and proteinase 3 (PR3). Significantly increased levels of all the analytes were observed in the patients’ samples, except for lactoferrin. In the RUXO patient group, only lipocalin was increased (Fig. 3A and Supplementary Figure 2B), however, the detection was performed only in 2 or 3 RUXO patient samples (depending on the method ELISA or LUMINEX). The *p*-values are displayed in Fig. 3B.

When isolated neutrophils were incubated in complete medium overnight, increased levels of MMP8 and lipocalin were noted in the supernatants (Fig. 3C). Similarly, upon stimulation with LPS and zymosan, increased MMP8 and lipocalin were detected in STAT1 GOF neutrophil cultures (Fig. 3D).

### Peripheral STAT1 GOF Neutrophils Are Activated

Next, we analyzed the STAT1 GOF neutrophil phenotype by flow cytometry. Signs of activation were detected, specifically, increased CD16 and HLA-DR, and reduced L-selectin CD62L on patients, as well as on RUXO-treated patient neutrophils (Fig. 3E). Decreased surface CD62L was mirrored by increased serum levels of soluble CD62 ligand (sCD62L), implying an enhanced shedding of CD62L, a characteristic feature of neutrophil activation (Fig. 3F).

To expand the characterization of neutrophil activation status, we examined serum levels of S100A8/A9 and chemokines involved in neutrophil activation and migration, CXCL1, and IL-8. All analytes were increased in STAT1 GOF patients, regardless of RUXO treatment (Fig. 3G). Accordingly, increased expression of CXCR2, a receptor for IL-8 and CXCL1, was detected on patient neutrophils (Fig. 3I).

### Suppressive and Immature Neutrophil Subsets Are Expanded in STAT1 GOF

**Fig. 4:**
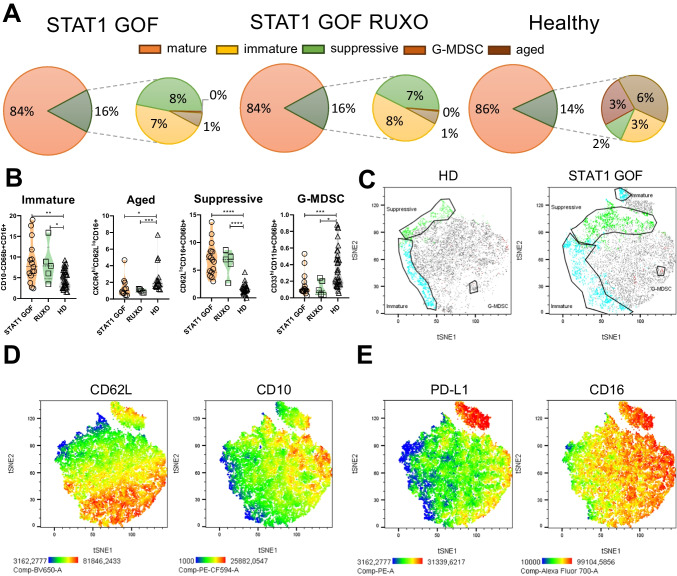
STAT1 GOF neutrophil subpopulations. **A** Radar plots of neutrophil subsets. **B** Quantification of neutrophil subsets in STAT1 GOF (*n*=15), RUXO (*n*=5), and HD (*n*=33) detected by flow cytometry. **C** Neutrophil subsets depicted as manually gated populations overlaid into t-SNE plots; green—suppressive, blue—immature, pink—G-MDSC. **D** t-SNE visualization of neutrophil phenotype. t-SNE plots of 5 STAT1 GOF patients and 5 HDs showing expression of CD62L and CD10 and **E** PD-L1 and CD16. HD, healthy donors; RUXO, ruxolitinib-treated patients; G-MDSC, granulocyte-myeloid-derived suppressive cells; t-SNE, t-distributed stochastic neighbor embedding. Values are standardized and expressed as median values. Statistical analyses were performed using paired *t*-tests. Values of *p*<0.05 (*), *p*<0.01 (**), *p*<0.001 (***), and *p*<0.0001 (****) were considered statistically significant

In the next step, we assessed the proportion of neutrophil subsets—the mature (CD10+), immature (CD10-), suppressive (CD62L^low^CD16^hi^), aged (CXCR4+CD62L−), and granulocyte-myeloid derived suppressive cells (G-MDSC, CD33+CD11b+). STAT1 GOF patients displayed significant alterations in their distribution. The mature, aged, and G-MDSC neutrophils were decreased, while the immature and suppressive subsets were expanded (Fig. 4A–C). RUXO neutrophils did not differ significantly from the untreated patient cells (Fig. 4A, B). As immature neutrophils are released from the bone marrow upon CXCL2 exposure, we analyzed the expression of CXCL2 receptor (CXCR2) on patient neutrophils, which was increased (Fig. 3I).

As expected, suppressive neutrophils had diminished expression of CD62L, and immature neutrophils lacked CD10 marker (Fig. 4D). Additionally, a profound lack of PD-L1 on immature neutrophils and a reduction of CD16 on both immature and suppressive neutrophils was seen (Fig. 4E).

### Phagocytosis and ROS Production in Unaltered, but Pro-inflammatory Cytokine Production Is Enhanced in STAT1 GOF Neutrophils

**Fig. 5:**
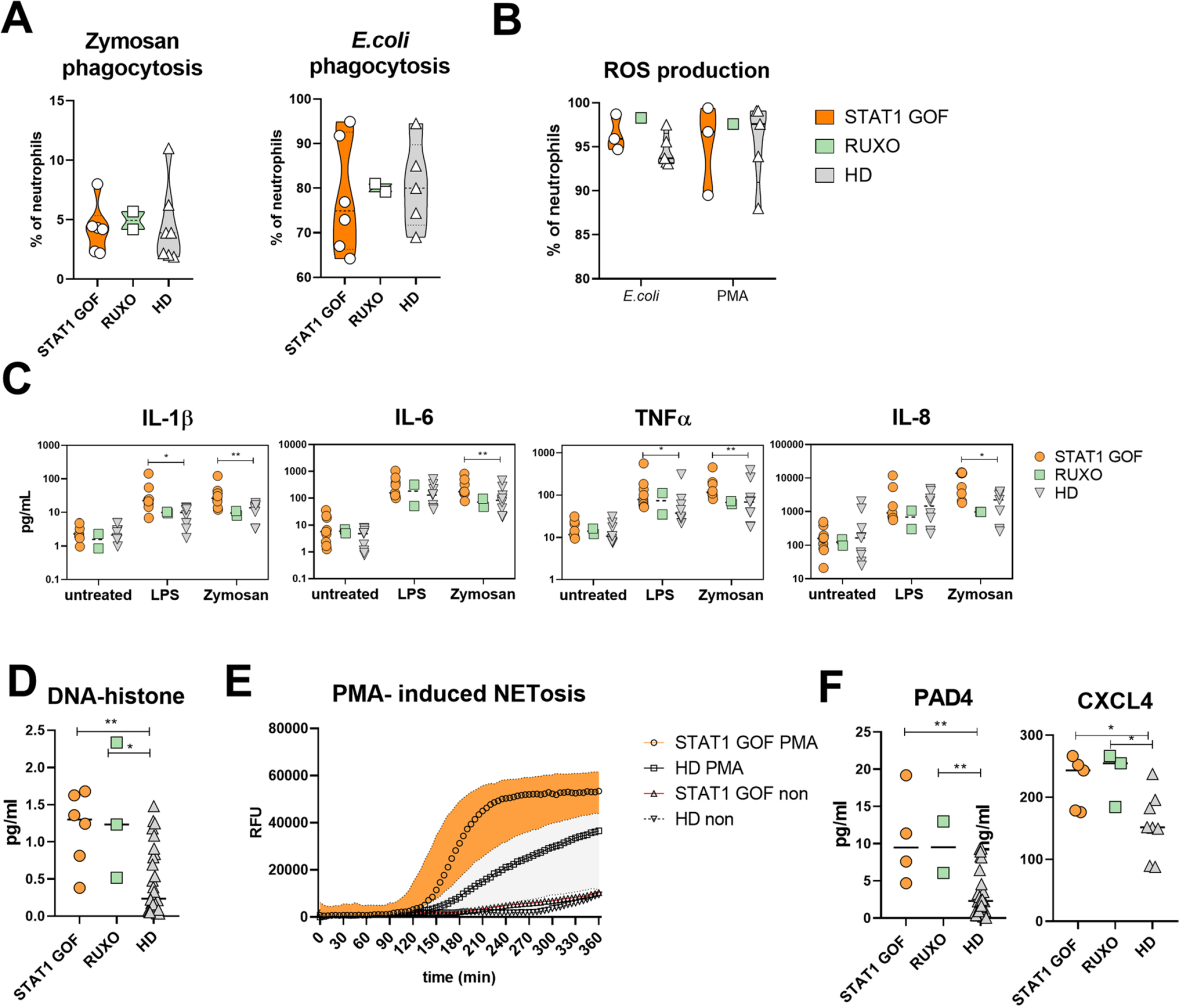
STAT1 GOF neutrophil features. **A** Zymosan and E.coli phagocytosis by neutrophils in STAT1 GOF (*n*=6), RUXO (*n*=2), and HD (*n*=7) detected by flow cytometry. **B** Reactive oxygen species production by neutrophils in STAT1 GOF (*n*=3), RUXO (*n*=1), and HD (*n*=5) detected by flow cytometry. **C** Cytokine production by isolated neutrophils in STAT1 GOF (*n*=9) and HD (*n*=8) upon overnight LPS and zymosan exposure detected by Luminex. **D** Serum levels of DNA-histone complexes detected in STAT1 GOF (*n*=6), RUXO (*n*=3), and HD (*n*=28) detected by ELISA. **E** DNA release from neutrophils upon PMA stimulation in STAT1 GOF (*n*=3) and HD (*n*=3). **F** Serum levels of PAD4 and CXCL4 in STAT1 GOF (*n*=6), RUXO (*n*=3), and HD (*n*=33) detected by ELISA or Luminex, respectively. HD, healthy donors; RUXO, ruxolitinib-treated patients; LPS, lipopolysaccharide; PMA, phorbol 12-myristate 13-acetate; PAD4, peptidyl arginine deiminase 4. Values are standardized and expressed as median values. Statistical analyses were performed using paired *t*-tests. Values of *p*<0.05 (*), *p*<0.01 (**), *p*<0.001 (***), and *p*<0.0001 (****) were considered statistically significant

Functional assessment of STAT1 GOF neutrophils revealed neither any alteration of their ability to phagocyte zymosan and *E. coli,* nor their ability to produce reactive oxygen species (ROS) (Fig. 5A, B). Using LUMINEX, the cytokine production of isolated STAT1 GOF neutrophils stimulated with LPS or zymosan was assessed. Patient neutrophils displayed significantly higher production of IL-1β, IL-6, and TNFα (Fig. 5C).

### NETosis-Related Products in STAT1 GOF Patients Are Elevated

Increased serum levels of degranulation markers, such as MPO or NE, suggested enhanced NETosis in the patients (Fig. 3A). Moreover, increased level of DNA-histone complexes, another NETosis-related marker, was found in the patient sera (Fig. 5D). Upon phorbol myristate acetate stimulation, the patient cells released more NETs than HD (Fig. 5E). Additionally, increased levels of peptidyl arginine deiminase 4 (PAD4), an enzyme involved in first steps of NETs formation, were detected (Fig. 5F), as well as increased CXCL4, a chemokine engaged in NET formation [23].

### Circulating Platelet-Neutrophil Aggregates Are Enhanced and Activated in STAT1 GOF

**Fig. 6:**
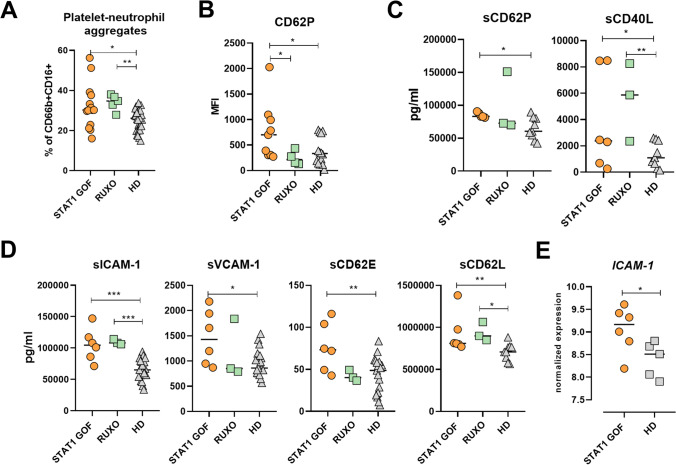
STAT1 GOF neutrophil interactions with platelets. **A** Quantification of platelet-neutrophil aggregates (PNAs) in STAT1 GOF (*n*=14), RUXO (*n*=5), and HD (*n*=29) detected by flow cytometry. **B** CD62P expression on PNAs in STAT1 GOF (*n*=9), RUXO (*n*=4), and HD (*n*=14) detected by flow cytometry. **C** Serum levels of CD62P and CD40L in STAT1 GOF (*n*=5), RUXO (*n*=3), and HD (*n*=9). **D** Serum levels of ICAM-1, VCAM-1, CD62E, and CD62L in STAT1 GOF (*n*=6), RUXO (*n*=3), and HD (*n*=20) **E.** *ICAM-1* normalized expression in in STAT1 GOF (*n*=6) and HD (*n*=5) neutrophils detected by nanoString. HD, healthy donors; RUXO, ruxolitinib-treated patients; ICAM, intracellular adhesive molecule; VCAM, vascular cell adhesion protein. Values are standardized and expressed as median values. Statistical analyses were performed using paired *t*-tests. Values of *p*<0.05 (*), *p*<0.01 (**), *p*<0.001 (***), and *p*<0.0001 (****) were considered statistically significant

Lastly, to assess neutrophil interactions with platelets, we investigated the number of platelet-neutrophil aggregates (PNAs) in our cohort. PNAs were detected by flow cytometry as a co-expression of CD41 and CD16 on CD66b+CD16+ neutrophils. STAT1 GOF patients had elevated numbers of PNAs (Fig. 6A), regardless of RUXO treatment. The expression of P-selectin (CD62P), a platelet activation marker within PNAs, was higher in STAT1 GOF but not in RUXO samples (Fig. 6B). In STAT1 GOF, the increased surface expression was accompanied by higher serum levels of soluble CD62P (Fig. 6C). Moreover, levels of soluble CD40L, another platelet activation marker, were also increased in both STAT1 GOF and RUXO samples (Fig. 6C). Overall, these findings suggest that circulating PNAs in STAT1 patients are activated and RUXO has little effect on their formation.

Since platelets assist neutrophils in migration and adhesion, we also assessed serum levels of soluble adhesion molecules, sICAM-1, sVCAM-1, sCD62E, and sCD62L. All examined analytes were elevated in STAT1 GOF patients, but only sICAM-1 and sCD62L were increased in RUXO patients (Fig. 6D). Moreover, increased RNA levels of *ICAM-1* were detectable in STAT1 GOF neutrophils (Fig. 6E).

## Discussion

Despite increasing evidence of STAT1 signaling relevance in neutrophil functions, this cell population has not been explored in the context of STAT1 GOF CMC to date. In this work, we demonstrate that STAT1 GOF neutrophils are primed toward heightened inflammatory states, evidenced by their phenotype, enhanced activation, degranulation, NETosis, and production of pro-inflammatory cytokines. We report evidence of increased IFN signature and detect little effect of JAK inhibition on the alterations observed herein.

The role of JAK/STAT signaling in neutrophils is manifold, context-dependent, and complexly negatively regulated. Colony-stimulating factors, various cytokines, and IFNs regulate myeloid development, proliferation, and functions and help integrate early innate immune responses utilizing the STATs [6, 24, 25]. STAT1 is utilized by type I IFNs to exert antiapoptotic effects on neutrophils [26] and by type II IFNs to enhance phagocytosis [27]. STAT1-mediated signaling of IFNs also affect oxidative burst, neutrophil migration and increases NET formation by mature neutrophils [28]. STAT3 conveys signals from granulocyte-colony stimulating factors to mobilize neutrophils from bone marrow [29] and facilitates neutrophil chemotaxis and migration [24, 25, 30] on one hand; on the other hand, STAT3 negatively regulates IFN responses and has been proposed to block Toll-like receptor signaling [31]. A tightly regulated and finely tuned STAT1/STAT3 balance in response to various stimuli is, therefore, a major a determinant of cellular health, and its genetically driven impairment is likely detrimental to neutrophil performance. Neutrophil products or impaired functions are involved in immunopathology of several autoimmune diseases [32, 33]; therefore, we suggest that abnormally activated neutrophils may contribute to the autoimmune features of STAT1 GOF CMC.

Remarkably, neutrophils appear to be divergent from the JAK/STAT signaling pattern of other STAT1 GOF cell types. STAT1 phosphorylation, otherwise, characteristically increased in other STAT1 GOF-affected cell populations, such as monocytes, lymphocytes, or NK cells [17, 18, 22, 34], was not augmented in neutrophils. Despite the normal stimulated pSTAT1 levels, elevated expression of IFN-stimulated genes in STAT1 GOF neutrophils was observed. An intuitive explanation could be that the increased IFNα serum level enhanced the basal pSTAT1, observed in our patient neutrophils, and, consequently, decreased their ability to further overtly activate STAT1 upon additional *ex vivo* stimulation. However, contrary to this notion, unincreased serum IFNα level was previously reported in four patients [35]. Therefore, alternative explanations must be sought.

Interestingly, peripheral blood mononuclear cells from STAT1 GOF patients were shown to exhibit increased IFN signature [36], and mechanistically, the upregulated expression of IFN stimulated genes was demonstrated to be governed by epigenetic rewiring in STAT1 GOF [35]. This corresponds with our detection of increased IFN response in neutrophils. Translating well into the clinical manifestation, enhanced IFN signaling plays a role in promotion and advancement of autoimmune diseases [37–39], and in fact, STAT1 GOF manifestations have been likened to type I interferonopathies [40]. These patients display persistent autoinflammation and autoimmune phenomena, such as SLE, autoimmune cytopenias, autoimmune thyroid disease, T1D, and much like the STAT1 GOF patients. Similar complications are sometimes experienced by receivers of recombinant IFNα [41, 42].

Autoimmunity is, however, a complex process, and we hypothesize that STAT1 GOF neutrophils corroborate its genesis by multiple mechanisms, influencing both the innate and adaptive immune operations [17, 18, 22, 43, 44]. For instance, molecules released by neutrophils, such as MPO and NE, were increased in the patient sera, and these have important roles in triggering proinflammatory responses [45]. Enhanced production of NETs and ROS, detected by the STAT1 GOF neutrophils, has been reported to exaggerate classic autoimmune diseases, such as SLE, rheumatoid arthritis, systemic sclerosis, or T1D [46–49]. Moreover, we recently reported that monocytes and dendritic cells of STAT1 GOF patients exhibit hyperactivated features with increased production of pro-inflammatory cytokines [17, 18], which may be, at least in part, due to their interplay with neutrophils [50–52].

Neutrophils, once considered a uniform population, are now appreciated for their phenotypical and physical diversity and plasticity, both maturation and activation-dependent. However, a uniform definition of individual subpopulations is lacking, and the understanding of their specific roles is limited. Nevertheless, the heterogenous identity of neutrophils and its bias from healthy states has already been associated with numerous autoimmune diseases [53]. For instance, immature CD10- neutrophils, noted to be increased in the STAT1 GOF here, were found expanded in patients with immune systemic vasculitides [54]. On the other hand, it was the expansion of the mature CD10+ forms that positively correlated with lupus damage index in SLE [55]. Interestingly, the G-MDSC subset, known from autoimmune pathologies and cancer immunology to actively suppress T cell responses [56–58], was found diminished in this cohort of STAT1 GOF patients, presenting another possible module of immunoregulatory failure.

Neutrophils interact with platelets via adhesion and formation of PNAs. PNAs trigger neutrophil activation, degranulation, and NET formation and facilitate their recruitment and migration into the site of inflammation [59–67]. Elevated counts of PNAs have been reported in autoimmunity, such as in T1D [68] and psoriasis [69]. Having found increased PNAs in the STAT1 GOF patients, we suggest that they may constitute another relevant driver of the STAT1 GOF autoimmunity.

Furthermore, the interaction between platelets and neutrophils also represents a driving force in various vascular diseases, such as aortic aneurysm (AA) [70], and large vessel aneurysms are one of the unexplained symptoms of STAT1 GOF CMC, present in approximately 6 % of patients [2, 71]. AA patients have increased leukocyte-platelet aggregates, and inhibition of platelet activation factors, such as CD62P and CD40-CD40L axis, lowers AA incidence and the risk of AA rupture [72–74]. Moreover, IFNγ was suggested to stimulate macrophageal release of MMP, causing tissue destruction resulting in AA formation in mice [75], and we observed that MMP was produced in abundance by STAT1 GOF neutrophils. Taken together, neutrophils’ role in aneurysm development in STAT1 GOF CMC should be explored further as it may help elucidate immune mechanisms contributing to aneurysm formation in general.

Finally, the JAK1/2 inhibitor ruxolitinib, which blocks the JAK kinase upstream from STATs, has been successfully used to ameliorate CMC and autoimmunity in several STAT1 GOF patients [22, 76]. Importantly, it failed in others [77]. Here, ruxolitinib had little effect on the neutrophil phenotype, as assessed by cytokine release, degranulation, or transcriptomic profile. In fact, in several experiments, it even amplified the disparity of STAT1 GOF and HD neutrophils. Therefore, we suggest that (a) the proinflammatory alterations of STAT1 GOF neutrophils are principally not likely intrinsically STAT1-driven, (b) neutrophils contribute to the IFN-governed immunopathology via other STAT1-independent mechanisms, and (c) this STAT1-independent mechanism may entail the failure of JAK inhibition in some STAT1 GOF patients, as the proinflammatory bias of neutrophils remains unaffected by JAKinib.

We acknowledge that this study is constrained by several limitations. These include the relatively small size of the studied cohort, as well as its heterogeneity. As genotype-specific molecular fingerprints likely affect the resulting STAT1 GOF clinical phenotypes [78], the cumulative study of neutrophils from patients with various mutations and varied clinical severity may not accurately capture the mutation-specific patterns and introduce an unwanted bias. Moreover, we did not ascertain the extent to which the observed phenomena are cell-intrinsic or secondary to the overall proinflammatory milieu of STAT1 GOF. Also, as in any study on *ex vivo* cells, the many potential confounding variables must be acknowledged, such as the effect of therapy, nutrition, ongoing infections, or other concomitant illnesses. To address these limitations and assess the effect of STAT1 GOF mutation on human neutrophils void of such influences, we suggest that studies on i*n-vitro* models should be undertaken and correlated to the hereby presented results. Finally, studies on tissue-recruited neutrophils are necessary to correlate our observations from peripheral blood with the site-specific microenvironments.

To conclude, this study demonstrates that neutrophils in STAT1 GOF CMC are altered in phenotype and functions. They are highly activated and have strong propensity for degranulation, NETosis, and platelet-neutrophil aggregation. They display immature phenotype and marked inflammatory bias with pronounced IFN signature. Ligand-induced STAT1 phosphorylation is not increased in STAT1 GOF neutrophils, and treatment with JAK1/2 inhibitor has little effect on the observed neutrophil alterations.

In summary, this work provides first evidence that neutrophils may contribute to the autoimmune phenomena and co-orchestrate the vascular complications in STAT1 GOF CMC. As such, further exploration of neutrophil involvement in STAT1 GOF immunopathology is fully warranted.

## Supplementary Information

ESM 1 (PDF 598 kb)

## Data Availability

The datasets generated during and/or analyzed during the current study are available from the corresponding author on reasonable request.

## References

[1] Liu L, Okada S, Kong XF, Kreins AY, Cypowyj S, Abhyankar A, Toubiana J, Itan Y, Audry M, Nitschke P (2011). Gain-of-function human STAT1 mutations impair IL-17 immunity and underlie chronic mucocutaneous candidiasis. J Exp Med..

[2] Toubiana J, Okada S, Hiller J, Oleastro M, Gomez ML, Becerra JCA, Ouachée-Chardin M, Fouyssac F, Girisha KM, Etzioni A (2016). Heterozygous STAT1 gain-of-function mutations underlie an unexpectedly broad clinical phenotype. Blood..

[3] Depner M, Fuchs S, Raabe J, Frede N, Glocker C, Doffinger R, Gkrania-Klotsas E, Kumararatne D, Atkinson TP, Schroeder HW (2016). The extended clinical phenotype of 26 patients with chronic mucocutaneous candidiasis due to gain-of-function mutations in STAT1. J Clin Immunol..

[4] van de Veerdonk FL, Plantinga TS, Hoischen A, Smeekens SP, Joosten LAB, Gilissen C, Arts P, Rosentul DC, Carmichael AJ, Smits-van der Graaf CAA (2011). STAT1 mutations in autosomal dominant chronic mucocutaneous candidiasis. N Engl J Med..

[5] O’Shea JJ, Plenge R (2012). JAK and STAT Signaling Molecules in immunoregulation and immune-mediated disease. Immunity..

[6] Hu X, Li J, Fu M, Zhao X, Wang W (2021). The JAK/STAT signaling pathway: from bench to clinic. Signal Transduct Target Ther..

[7] Aittomäki S, Pesu M (2014). Therapeutic targeting of the JAK/STAT pathway. Basic Clin Pharmacol Toxicol..

[8] Dupuis S, Jouanguy E, Al-Hajjar S, Fieschi C, Zaid Al-Mohsen I, Al-Jumaah S, Yang K, Chapgier A, Eidenschenk C, Eid P (2003). Impaired response to interferon-alpha/beta and lethal viral disease in human STAT1 deficiency. Nat Genet..

[9] Takayanagi H, Kim S, Koga T, Taniguchi T (2005). Stat1-mediated cytoplasmic attenuation in osteoimmunology. J Cell Biochem..

[10] Kovacic B, Stoiber D, Moriggl R, Weisz E, Ott RG, Kreibich R, Levy DE, Beug H, Freissmuth M, Sexl V (2006). STAT1 acts as a tumor promoter for leukemia development. Cancer Cell..

[11] Najjar I, Deglesne P-A, Schischmanoff PO, Fabre EE, Boisson-Dupuis S, Nimmerjahn F, Bornkamm GW, Dusanter-Fourt I, Fagard R (2010). STAT1-dependent IgG cell-surface expression in a human B cell line derived from a STAT1-deficient patient. J Leukoc Biol..

[12] Zimmerman O, Olbrich P, Freeman AF, Rosen LB, Uzel G, Zerbe CS, Rosenzweig SD, Kuehn HS, Holmes KL, Stephany D (2019). STAT1 gain-of-function mutations cause high total STAT1 levels with normal dephosphorylation. Front Immunol..

[13] Zheng J, van de Veerdonk FL, Crossland KL, Smeekens SP, Chan CM, Al Shehri T, Abinun M, Gennery AR, Mann J, Lendrem DW (2015). Gain-of-function STAT1 mutations impair STAT3 activity in patients with chronic mucocutaneous candidiasis (CMC). Eur J Immunol..

[14] Rodero MP, Crow YJ (2016). Type I interferon-mediated monogenic autoinflammation: The type I interferonopathies, a conceptual overview. J Exp Med..

[15] Crow YJ (2011). Type I interferonopathies: a novel set of inborn errors of immunity. Ann N Y Acad Sci..

[16] Crow YJ, Lebon P, Casanova JL, Gresser I (2018). A Brief Historical perspective on the pathological consequences of excessive type I interferon exposure in vivo. J Clin Immunol..

[17] Bloomfield M, Zentsova I, Milota T, Sediva A, Parackova Z. Immunoprofiling of monocytes in STAT1 gain-offunction chronic mucocutaneous candidiasis. Front Immunol. 2022;13:1–14. 10.3389/FIMMU.2022.983977. 10.3389/fimmu.2022.983977 PMC9510987 36172362

[18] Parackova Z, Zentsova I, Vrabcova P, Sediva A, Bloomfield M (2022). Aberrant tolerogenic functions and proinflammatory skew of dendritic cells in STAT1 gain-of-function patients may contribute to autoimmunity and fungal susceptibility. Clin Immunol..

[19] Welte K, Zeidler C, Dale DC (2006). Severe congenital neutropenia. Semin Hematol..

[20] Németh T, Sperandio M, Mócsai A (2020). Neutrophils as emerging therapeutic targets. Nat Rev Drug Discov..

[21] Pettersen EF, Goddard TD, Huang CC, Meng EC, Couch GS, Croll TI, Morris JH, Ferrin TE (2021). UCSF ChimeraX: Structure visualization for researchers, educators, and developers. Protein Sci..

[22] Bloomfield M, Kanderová V, Paračková Z, Vrabcová P, Svatoň M, Froňková E, Fejtková M, Zachová R, Rataj M, Zentsová I (2018). Utility of ruxolitinib in a child with chronic mucocutaneous candidiasis caused by a novel STAT1 gain-of-function mutation. J Clin Immunol..

[23] Matsumoto K, Yasuoka H, Yoshimoto K, Suzuki K, Takeuchi T (2021). Platelet CXCL4 mediates neutrophil extracellular traps formation in ANCA-associated vasculitis. Sci Rep..

[24] Panopoulos AD, Zhang L, Snow JW, Jones DM, Smith AM, El Kasmi KC, Liu F, Goldsmith MA, Link DC, Murray PJ (2006). STAT3 governs distinct pathways in emergency granulopoiesis and mature neutrophils. Blood..

[25] Zhang H, Nguyen-Jackson H, Panopoulos AD, Li HS, Murray PJ, Watowich SS (2010). STAT3 controls myeloid progenitor growth during emergency granulopoiesis. Blood..

[26] Sakamoto E, Hato F, Kato T, Sakamoto C, Akahori M, Hino M, Kitagawa S (2005). Type I and type II interferons delay human neutrophil apoptosis via activation of STAT3 and up-regulation of cellular inhibitor of apoptosis 2. J Leukoc Biol..

[27] Perussia B, Kobayashi M, Rossi ME, Anegon I, Trinchieri G (1987). Immune interferon enhances functional properties of human granulocytes: role of Fc receptors and effect of lymphotoxin, tumor necrosis factor, and granulocyte-macrophage colony-stimulating factor. J Immunol..

[28] Pylaeva E, Lang S, Jablonska J (2016). The essential role of type I interferons in differentiation and activation of tumor-associated neutrophils. Front Immunol..

[29] McLemore ML, Grewal S, Liu F, Archambault A, Poursine-Laurent J, Haug J, Link DC (2001). STAT-3 activation is required for normal G-CSF-dependent proliferation and granulocytic differentiation. Immunity..

[30] Semerad CL, Liu F, Gregory AD, Stumpf K, Link DC (2002). G-CSF is an essential regulator of neutrophil trafficking from the bone marrow to the blood. Immunity..

[31] Wang W-B, Levy DE, Lee C-K (2011). STAT3 negatively regulates type I IFN-mediated antiviral response. J Immunol..

[32] Kaplan MJ (2013). Role of neutrophils in systemic autoimmune diseases. Arthritis Res Ther..

[33] Németh T, Mócsai A (2012). The role of neutrophils in autoimmune diseases. Immunol Lett..

[34] Tabellini G, Vairo D, Scomodon O, Tamassia N, Ferraro RM, Patrizi O, Gasperini S, Soresina A, Giardino G, Pignata C (2017). Impaired natural killer cell functions in patients with signal transducer and activator of transcription 1 (STAT1) gain-of-function mutations. J Allergy Clin Immunol..

[35] Kaleviste E, Saare M, Leahy TR, Bondet V, Duffy D, Mogensen TH, Jørgensen SE, Nurm H, Ip W, Davies EG (2019). Interferon signature in patients with STAT1 gain-of-function mutation is epigenetically determined. Eur J Immunol..

[36] Stellacci E, Moneta GM, Bruselles A, Barresi S, Pizzi S, Torre G, De Benedetti F, Tartaglia M, Insalaco A (2019). The activating p.Ser466Arg change in STAT1 causes a peculiar phenotype with features of interferonopathies. Clin Genet..

[37] Psarras A, Emery P, Vital EM (2017). Type I interferon–mediated autoimmune diseases: pathogenesis, diagnosis and targeted therapy. Rheumatology..

[38] Di Domizio J, Cao W (2013). Fueling autoimmunity: type I interferon in autoimmune diseases. Expert Rev Clin Immunol..

[39] Niewold TB. Type I interferon in human autoimmunity. Front Immunol. 2014;5:1–2. 10.3389/FIMMU.2014.00306. 10.3389/fimmu.2014.00306 PMC4074699 25071767

[40] Okada S, Asano T, Moriya K, Boisson-Dupuis S, Kobayashi M, Casanova JL, Puel A (2020). Human STAT1 gain-of-function heterozygous mutations: chronic mucocutaneous candidiasis and type I interferonopathy. J Clin Immunol..

[41] Tolaymat A, Leventhal B, Sakarcan A, Kashima H, Monteiro C (1992). Systemic lupus erythematosus in a child receiving long-term interferon therapy. J Pediatr..

[42] Crow YJ, Stetson DB (2022). The type I interferonopathies: 10 years on. Nat Rev Immunol..

[43] Jaeger BN, Donadieu J, Cognet C, Bernat C, Ordoñez-Rueda D, Barlogis V, Mahlaoui N, Fenis A, Narni-Mancinelli E, Beaupain B (2012). Neutrophil depletion impairs natural killer cell maturation, function, and homeostasis. J Exp Med..

[44] Micheletti A, Costantini C, Calzetti F, Camuesco D, Costa S, Tamassia N, Cassatella MA (2013). Neutrophils promote 6-sulfo LacNAc+ dendritic cell (slanDC) survival. J Leukoc Biol..

[45] Yang D, de la Rosa G, Tewary P, Oppenheim JJ (2009). Alarmins link neutrophils and dendritic cells. Trends Immunol..

[46] Elloumi N, Ben Mansour R, Marzouk S, Mseddi M, Fakhfakh R, Gargouri B, Masmoudi H, Lassoued S (2017). Differential reactive oxygen species production of neutrophils and their oxidative damage in patients with active and inactive systemic lupus erythematosus. Immunol Lett..

[47] Fresneda Alarcon M, McLaren Z, Wright HL. Neutrophils in the pathogenesis of rheumatoid arthritis and systemic lupus erythematosus: same foe different M.O. Front Immunol. 2021;12:1–22. 10.3389/FIMMU.2021.649693. 10.3389/fimmu.2021.649693 PMC7969658 33746988

[48] Didier K, Giusti D, Le Jan S, Terryn C, Muller C, Pham BN, Le Naour R, Antonicelli FD, Servettaz A (2020). Neutrophil extracellular traps generation relates with early stage and vascular complications in ssystemic sclerosis. J Clin Med..

[49] Kundu S, Ghosh P, Datta S, Ghosh A, Chattopadhyay S, Chatterjee M (2012). Oxidative stress as a potential biomarker for determining disease activity in patients with rheumatoid arthritis. Free Radic Res..

[50] Prame Kumar K, Nicholls AJ, Wong CHY (2018). Partners in crime: neutrophils and monocytes/macrophages in inflammation and disease. Cell Tissue Res..

[51] Schuster S, Hurrell B, Tacchini-Cottier F (2013). Crosstalk between neutrophils and dendritic cells: a context-dependent process. J Leukoc Biol..

[52] Parackova Z, Zentsova I, Vrabcova P, Klocperk A, Sumnik Z, Pruhova S, Petruzelkova L, Hasler R, Sediva A (2020). Neutrophil extracellular trap induced dendritic cell activation leads to Th1 polarization in type 1 diabetes. Front Immunol..

[53] Soehnlein O, Steffens S, Hidalgo A, Weber C (2017). Neutrophils as protagonists and targets in chronic inflammation. Nat Rev Immunol..

[54] Grieshaber-Bouyer R, Nigrovic PA. Neutrophil heterogeneity as therapeutic opportunity in immune-mediated disease. Front Immunol. 2019;10:1–13. 10.3389/FIMMU.2019.00346. 10.3389/fimmu.2019.00346 PMC6409342 30886615

[55] Mistry P, Nakabo S, O’Neil L, Goel RR, Jiang K, Carmona-Rivera C, Gupta S, Chan DW, Carlucci PM, Wang X (2019). Transcriptomic, epigenetic, and functional analyses implicate neutrophil diversity in the pathogenesis of systemic lupus erythematosus. Proc Natl Acad Sci U S A..

[56] Crook KR, Liu P (2014). Role of myeloid-derived suppressor cells in autoimmune disease. World J Immunol..

[57] Yin B, Ma G, Yen C-Y, Zhou Z, Wang GX, Divino CM, Casares S, Chen S-H, Yang W-C, Pan P-Y (2010). Myeloid-derived suppressor cells prevent type 1 diabetes in murine models. J Immunol..

[58] Li M, Zhu D, Wang T, Xia X, Tian J, Wang S (2018). Roles of myeloid-derived suppressor cell subpopulations in autoimmune arthritis. Front Immunol..

[59] Perdomo J, Leung HHL, Ahmadi Z, Yan F, Chong JJH, Passam FH, Chong BH (2019). Neutrophil activation and NETosis are the major drivers of thrombosis in heparin-induced thrombocytopenia. Nat Commun..

[60] Lisman T (2018). Platelet-neutrophil interactions as drivers of inflammatory and thrombotic disease. Cell Tissue Res..

[61] Bdeir K, Gollomp K, Stasiak M, Mei J, Papiewska-Pajak I, Zhao G, Worthen GS, Cines DB, Poncz M, Kowalska MA (2017). Platelet-specific chemokines contribute to the pathogenesis of acute lung injury. Am J Respir Cell Mol Biol..

[62] Zucoloto AZ, Jenne CN. Platelet-neutrophil interplay: insights into neutrophil extracellular trap (NET)-driven coagulation in infection. 2019;6:85. https://pubmed.ncbi.nlm.nih.gov/31281822/. Accessed 9 Feb 2022. 10.3389/fcvm.2019.00085 PMC6595231 31281822

[63] Jenne CN, Kubes P (2015). Platelets in inflammation and infection. Platelets..

[64] Kim SJ, Jenne CN (2016). Role of platelets in neutrophil extracellular trap (NET) production and tissue injury. Semin Immunol..

[65] Finsterbusch M, Schrottmaier WC, Kral-Pointner JB, Salzmann M, Assinger A (2018). Measuring and interpreting platelet-leukocyte aggregates. Platelets..

[66] Kral JB, Schrottmaier WC, Salzmann M, Assinger A (2016). Platelet interaction with innate immune cells. Transfus Med Hemotherapy..

[67] McDonald B, Urrutia R, Yipp BG, Jenne CN, Kubes P (2012). Intravascular neutrophil extracellular traps capture bacteria from the bloodstream during sepsis. Cell Host Microbe..

[68] Popp SK, Vecchio F, Brown DJ, Fukuda R, Suzuki Y, Takeda Y, Wakamatsu R, Sarma MA, Garrett J, Giovenzana A, et al. Circulating platelet-neutrophil aggregates characterize the development of type 1 diabetes in humans and NOD mice. JCI Insight. 2022;7:1–16. 10.1172/JCI.INSIGHT.153993. 10.1172/jci.insight.153993 PMC8855805 35076023

[69] Herster F, Bittner Z, Codrea MC, Archer NK, Heister M, Löffler MW, Heumos S, Wegner J, Businger R, Schindler M (2019). Platelets aggregate with neutrophils and promote skin pathology in psoriasis. Front Immunol..

[70] Schrottmaier WC, Mussbacher M, Salzmann M, Assinger A (2020). Platelet-leukocyte interplay during vascular disease. Atherosclerosis..

[71] Bierman-Chow S, Freeman AF, Holland SM, Lynch J, Cho HJ (2022). Cerebral aneurysm in three pediatric patients with STAT1 gain-of-function mutations. J Neurol..

[72] Allen N, Barrett TJ, Guo Y, Nardi M, Ramkhelawon B, Rockman CB, Hochman JS, Berger JS (2019). Circulating monocyte-platelet aggregates are a robust marker of platelet activity in cardiovascular disease. Atherosclerosis..

[73] Kusters PJH, Seijkens TTP, Beckers L, Lievens D, Winkels H, De Waard V, Duijvestijn A, Liljeqvist ML, Roy J, Daugherty A (2018). CD40L deficiency protects against aneurysm formation. Arterioscler Thromb Vasc Biol..

[74] Hannawa KK, Cho BS, Sinha I, Roelofs KJ, Myers DD, Wakefield TJ, Stanley JC, Henke PK, Upchurch GR (2006). Attenuation of experimental aortic aneurysm formation in P-selectin knockout mice. Ann N Y Acad Sci..

[75] Zhou H, Yan H, Cannon JL, Springer LE, Green JM, Pham CTN (2013). CD43-mediated IFN-γ production by CD8+ T cells promotes abdominal aortic aneurysm in mice. J Immunol..

[76] Deyà-Martínez A, Rivière JG, Roxo-Junior P, Ramakers J, Bloomfield M, Guisado Hernandez P, Blanco Lobo P, Abu Jamra SR, Esteve-Sole A, Kanderova V (2022). Impact of JAK inhibitors in pediatric patients with STAT1 gain of function (GOF) mutations—10 children and review of the literature. J Clin Immunol..

[77] Zimmerman O, Rösler B, Zerbe CS, Rosen LB, Hsu AP, Uzel G, Freeman AF, Sampaio EP, Rosenzweig SD, Kuehn HS, et al. Risks of ruxolitinib in STAT1 gain-of-function-associated severe fungal disease. Open Forum Infect Dis. 2017;4:1–5. 10.1093/OFID/OFX202. 10.1093/ofid/ofx202 PMC5714179 29226168

[78] Giovannozzi S, Demeulemeester J, Schrijvers R, Gijsbers R (2021). Transcriptional profiling of STAT1 gain-of-function reveals common and mutation-specific fingerprints. Front Immunol..

