# Supplementary material for: Neutrophils in STAT1 Gain-Of-Function Have a Pro-inflammatory Signature Which Is Not Rescued by JAK Inhibition
Source: J Clin Immunol. 2023 Jun 26;43(7):1640–59. doi: 10.1007/s10875-023-01528-1 (PMC10499747; doi:10.1007/s10875-023-01528-1)

Supplementary Figure titles and legends

Supplementary Figure 1: pSTATs analysis A. Mean fluorescence values of pSTAT1 and pSTAT3 in STAT1 GOF and HDs**. B.** Representative blots of STAT1 phosphorylation in STAT1 GOF patient and HD upon IFNα stimulation **C.** pSTAT1 **D.** total STAT1 and **D.** βactin

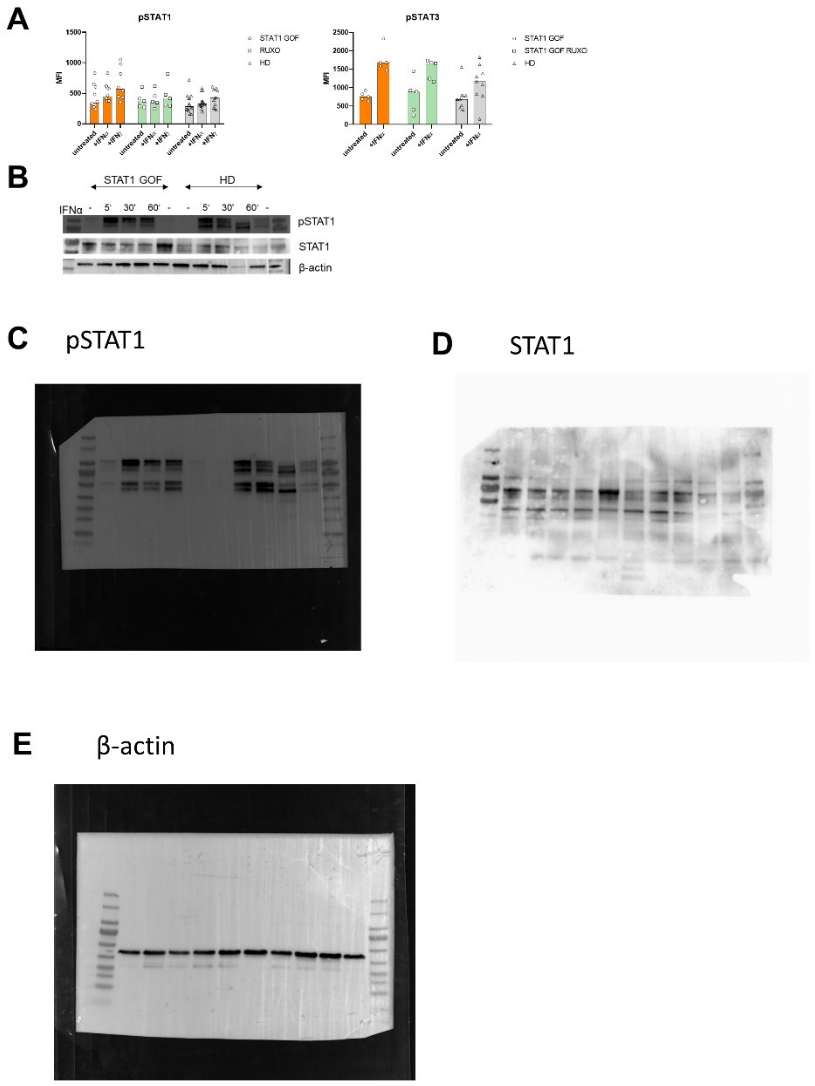

Supplementary Figure 2: STAT1 GOF neutrophil features. A. CXCL9 release by neutrophils upon zymosan stimulation and its combination with IFNα and IFNγ **B.** Serum levels of degranulation products

HD - healthy donors; ROS- reactive oxygen species; RUXO - ruxolitinib treated patients; PD-L1 – programmed cell death ligand 1; MPO – myeloperoxidase; NE- neutrophil elastase; MMP – matrix metaloproteinase 8; PR3 – proteinase 3. Values are standardized and expressed as median values. Statistical analyses were performed using paired t-tests. Values of p<0.05 (*), p<0.01 (**), p<0.001 (***), and p<0.0001 (****) were considered statistically significant.

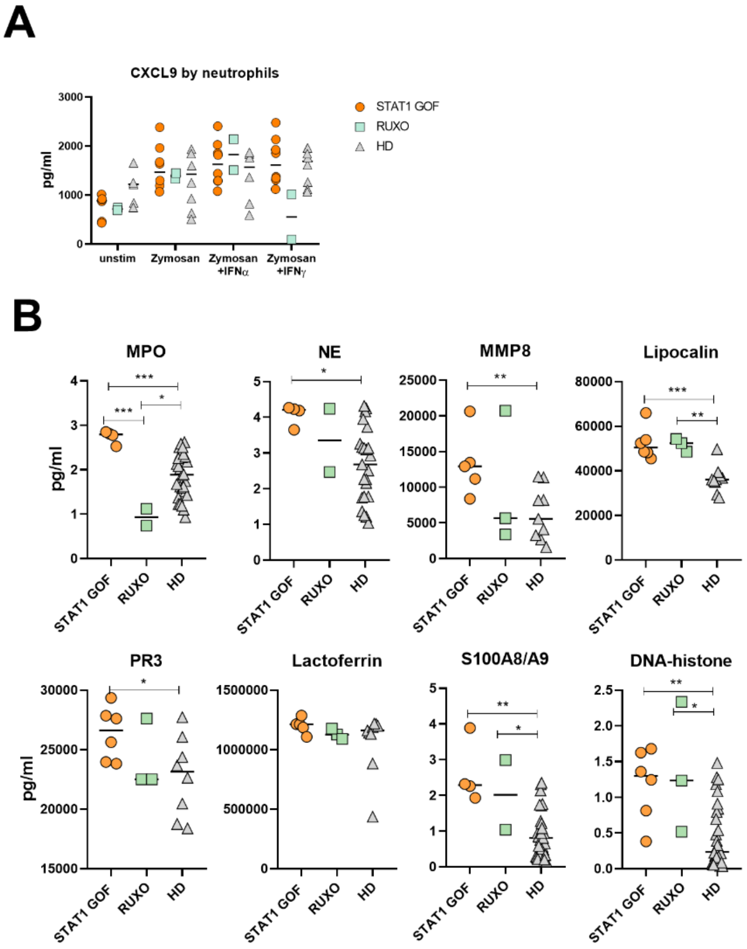

Supplementary Figure 3: RUXO-treated STAT1 GOF neutrophil transcriptomic analysis. Heatmap of differentially expressed genes (DEGs) in RUXO (n=2) and HD (n=5) neutrophils **B.** Volcano plot of up- and downregulated DEGs **C.** The most significant biological processes **D.** Volcano plot of terms in the Bioplanet_2019 gene set library. Each point represents a single term in the library, plotted by the corresponding odds ratio (x-position) and -log10 (p-value) (y-position) from the enrichment DEG results

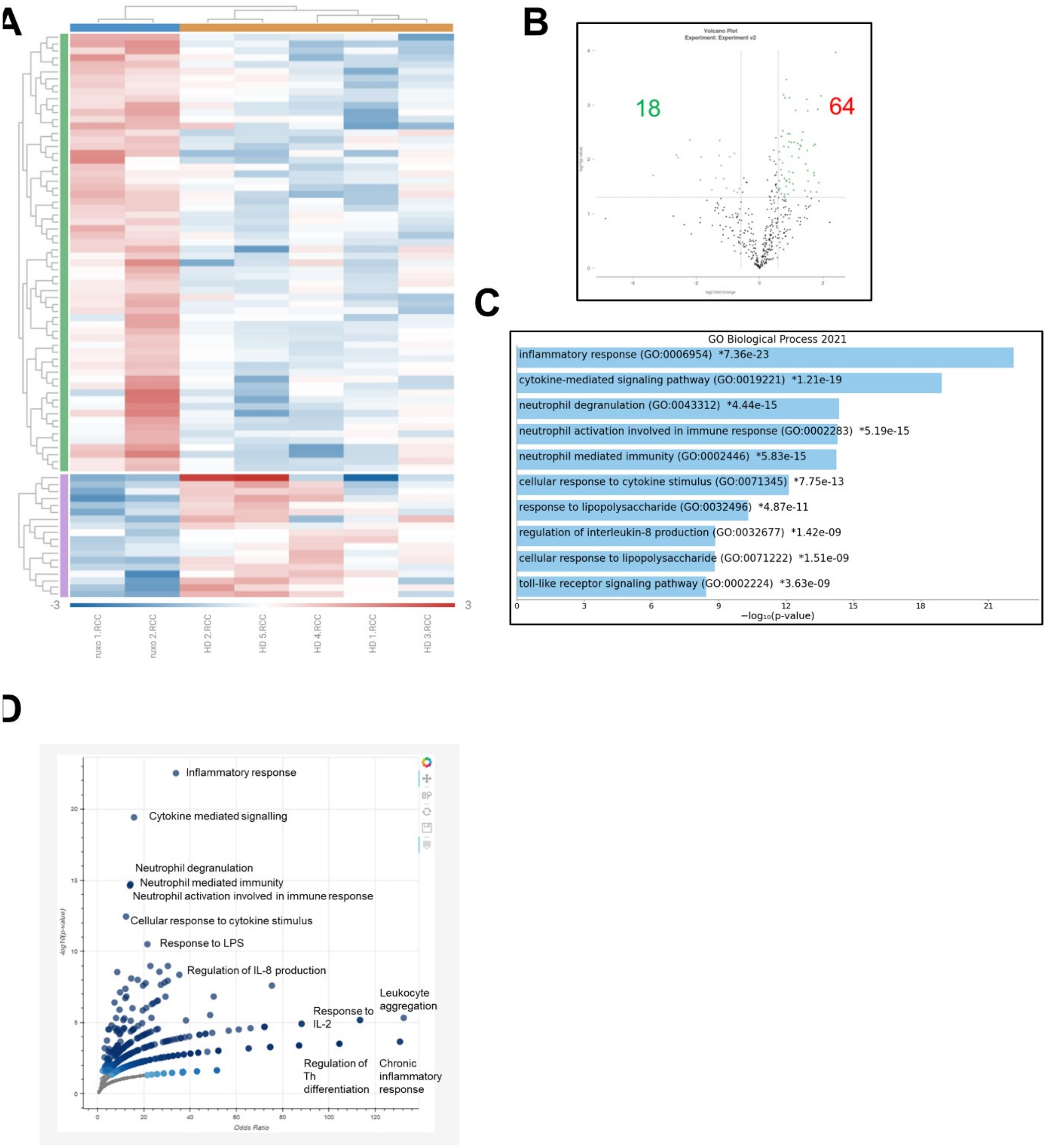

Supplement: Supplementary file 1 — (PDF 598 kb) [file 10875_2023_1528_MOESM1_ESM.pdf]
